# Graphene Dendrimer-stabilized silver nanoparticles for detection of methimazole using Surface-enhanced Raman scattering with computational assignment

**DOI:** 10.1038/srep32185

**Published:** 2016-08-30

**Authors:** Tawfik A. Saleh, Mutasem M. Al-Shalalfeh, Abdulaziz A. Al-Saadi

**Affiliations:** 1 Department of Chemistry; King Fahd University of Petroleum & Minerals, Dhahran 31261, Saudi Arabia

## Abstract

Graphene functionalized with polyamidoamine dendrimer, decorated with silver nanoparticles (G-D-Ag), was synthesized and evaluated as a substrate with surface-enhanced Raman scattering (SERS) for methimazole (MTZ) detection. Sodium borohydride was used as a reducing agent to cultivate silver nanoparticles on the dendrimer. The obtained G-D-Ag was characterized by using UV-vis spectroscopy, scanning electron microscope (SEM), high-resolution transmission electron microscope (TEM), Fourier-transformed infrared (FT-IR) and Raman spectroscopy. The SEM image indicated the successful formation of the G-D-Ag. The behavior of MTZ on the G-D-Ag as a reliable and robust substrate was investigated by SERS, which indicated mostly a chemical interaction between G-D-Ag and MTZ. The bands of the MTZ normal spectra at 1538, 1463, 1342, 1278, 1156, 1092, 1016, 600, 525 and 410 cm^−1^ were enhanced due to the SERS effect. Correlations between the logarithmical scale of MTZ concentrations and SERS signal intensities were established, and a low detection limit of 1.43 × 10^−12^ M was successfully obtained. The density functional theory (DFT) approach was utilized to provide reliable assignment of the key Raman bands.

Raman spectroscopy is based on the behavior of the inelastically scattered photons upon interaction with targeted molecules, and it has been recently becoming an attractive tool for various applications. The most challenging problem with Raman techniques is the nature of the weak scattering, which hinders its effective utilization, especially for low-detection limit targets. The surface-enhanced Raman scattering (SERS) approach, however, could provide a promising strategy to solve this problem. Moreover, given the noticeable advances in instrument technology, Raman spectroscopy has begun to compete with well-established traditional analytical techniques in terms of sensitivity and ease of use^1^.

In SERS, the targeted molecules are adsorbed from an aqueous solution onto nanoparticles that allow a charge transfer between analyte molecules and the particle surface, leading to an enhancement of the Raman signal^2^. Among the various commonly used types of materials to produce enhanced scattered Raman light are high-purity film-based substrates, which include metals settled on planar surfaces such as glass, quartz, and silicon wafers; or on nanoparticle-embedded surfaces such as silica beads and polystyrene^3^,^4^. SERS films can also be tuned somewhat to appropriate localized surface plasmon resonances by altering various parameters such as film thickness and deposition rate, with most thicknesses of metal being between 5–60 nm^5^. SERS substrates of colloidal silver or gold nanoparticles can consistently yield a large signal enhancement, explained by electromagnetic and/or chemical enhancement^6^.

Recently, SERS has been reported as a promising technique for quantitative and qualitative identifications of various targets^7^. It demonstrated the potential to impact the areas of analytical chemistry, biochemistry, forensics, environmental analysis, and trace analysis. The SERS approach exhibits a number of advantages for use in low-detection limit drug analysis when compared to other analytical techniques. Due to its ultra-sensitivity, SERS was used to detect trace organic and inorganic analytes in different media. For example, some organophosphorus compounds, such as methylparathiol and dimethoate, that exist in pesticides were identified at the nanogram level^8^. Because water molecules scatter weakly in Raman experiments, it has made the SERS approach an attractive choice to conduct useful characterization of samples^9^,^10^,^11^.

However, one of the most challenging tasks in developing an effective analytical SERS-based method is the fabrication of the right metal colloid substrate, such as silver, that can exhibit a hotspot within the nanoparticles and subsequently achieve extremely high enhancement^12^. Since it is required to have more nanoparticles to hook the targeted molecules, the use of a support to load the silver nanoparticles may control the agglomeration that diminishes the enhancement in SERS. Dendrimers, which represent a new class of polymeric nanoscale compounds, are promising candidates for SERS applications due to their homogeneous nature and unique tree-like structure. They have been found to be useful in the health industry, and in pharmaceutical and materials applications^13^. In addition, dendrimers are considered as one of the most appropriate encapsulating agents for the stabilization of metal nanoparticles (NPs), due to their large size and the presence of a unique three-dimensional architecture of the dendrons that prevents leaching of the NPs during the course of the reaction^14^. The polyamidoamine dendrimers are considered the favored choice for pharmaceutical applications, due to their regular structure, large size, and chemical versatility^15^.

Screening the literature reveals that several analytical procedures have been reported for the determination of a methimazole-based drug (also known as 1-methyl-2-mercapto-imidazole and tapazole), which is considered as an antihormone drug widely used to treat hyperthyroidism. These methods include molecularly imprinted biomimetic sensing^16^, fluorescence^17^, thin layer chromatography^18^, coulometry^19^, conductometry^20^, and high-performance liquid chromatography with ultraviolet detection^21^. To the best of our knowledge, no SERS attempts with the use of graphene dendrimeric-based substrates has been reported to detect low-concentration samples of methimazole (MTZ).

In this work we adopted graphene as a support, modified with a dendrimer, to allow controlled silver nanoparticles to be linked to its branches. The prepared graphene linked with dendrimer-stabilized silver nanoparticles (G-D-Ag) was then evaluated as a potential SERS substrate for MTZ detection.

## Experimental Procedure

### Chemicals and Materials

Methimazole (MTZ) “1-Methyl-2-imidazolethiol “(analytical standard, ≥99% purity), CAS number 60560, was purchased from Sigma-Aldrich. Silver nitrate (AgNO_3_, 99.8%), product number 30087, was purchased from BDH-Chemicals Ltd Poole England. Sodium borohydride (NaBH4), product number 63390, was purchased from Allied Signal. Ethylenediamine (≥99.5%), product number 03550, methyl acrylate (99%), CAS number 76778, thionyl chloride (SOCl_2_, ≥99%), product number 230464, and potassium bromide (KBr, ≥99%), product number 221864, were purchased from Sigma-Aldrich. Solutions were prepared with ultrapure water obtained from a water purification system (Ultra Clear™ Lab Water Systems, Siemens Water Technologies USA).

### Synthesis of graphene dendrimer silver composite

**Figure 1:**
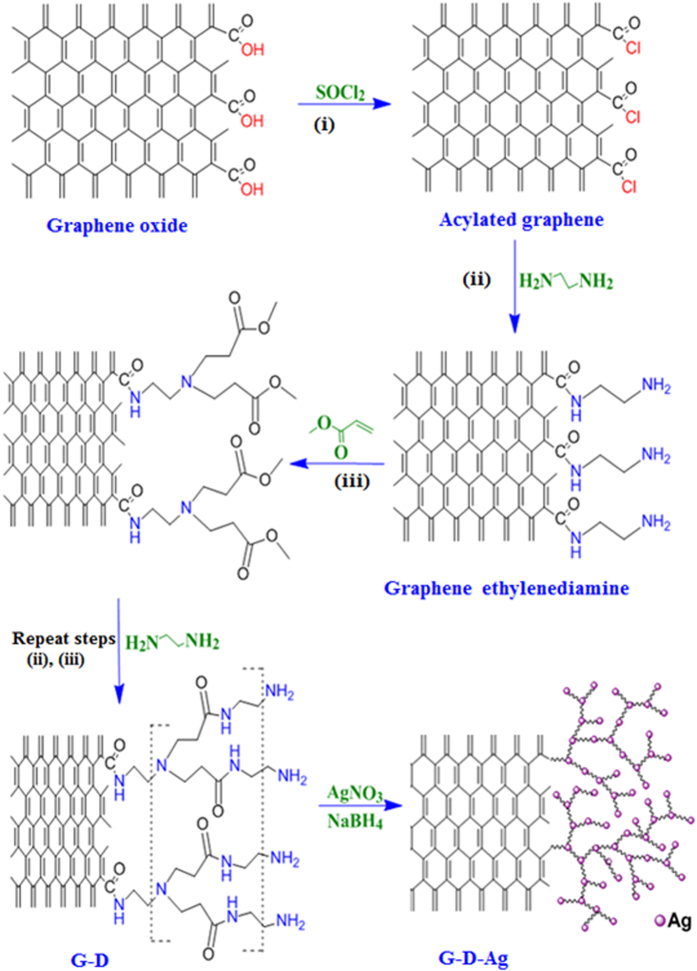
Illustration explaining the synthesis steps of the graphene- polyamidoamine dendrimer-silver G-D-Ag.

Figure 1 shows the preparation steps of dendrimer functionalization with silver. About 0.2 g of the prepared graphene nanosheets was dispersed in 20 ml of SOCl_2_ by sonication in an ultrasound bath for 30 min and stirred for 12 h at 60 °C; the mixture was then filtered. The obtained material was dried overnight at room temperature. Next, 10 ml of ethylenediamine was added to the solid product, the reaction mixture was sonicated for 3 h at 60 °C, and stirred for another 12 h at room temperature. The solid product was collected by centrifugation at 10000 rpm/min for 10 min and dried overnight at room temperature.

The last solid product was suspended in 10 ml methanol and was added dropwise to 25 ml of 1:4 methyl acrylate - methanol solution under stirring. The reaction mixture was treated in an ultrasonic bath at 60 °C for 2 hours and stirred for another 12 h at room temperature. The solid product was collected by centrifugation at 10000 rpm/min for 10 min and dried overnight at room temperature. Afterward, the obtained material was immersed in 10 ml methanol, and then a 1:1 mixture of 10 ml of ethylenediamine – methanol was added at a rate 1 drop/sec to the solution. The solution was placed in an ultrasonic bath at 50 °C for 5 h and stirred for another 10 h at room temperature. The solid product was collected by centrifugation and dried overnight at room temperature. The steps were repeated for methyl acrylate and ethylenediamine until reaching the third-generation. The third-generation polyamidoamine dendrimer on the graphene (G-D) presented a typical morphology when compared to the others obtained using higher dendrimer concentrations.

**Figure 2:**
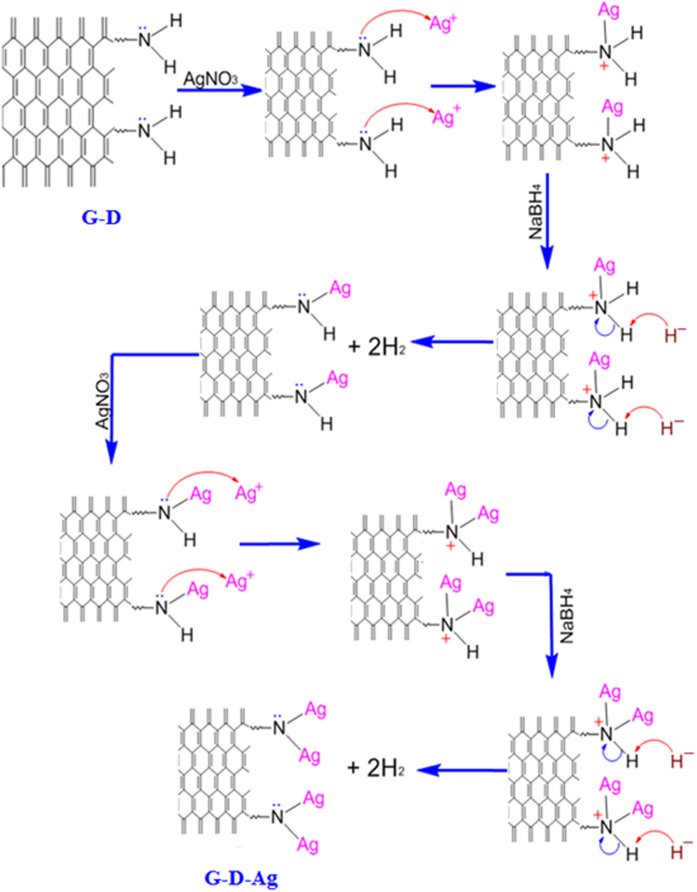
Mechanism of the stabilization of the AgNPs on the graphene through the dendrimer for the preparation of graphene- polyamidoamine dendrimer-silver (G-D-Ag).

The solid of this material was dispersed in 20 ml de-ionized water by sonication in an ultrasound bath for 10 min. Then, 10 ml of 0.2 M AgNO_3_ was added dropwise with the previously dispersed solid and the mixture was stirred for 1 hour. Then, 10 ml of a freshly prepared solution of NaBH_4_ was added to the solution and the solution was kept under stirring for another 5 h. Finally, the mixture was filtered, and the obtained material was washed with deionized water several times. The greenish yellow isolated solid was dried overnight at room temperature. The stabilization mechanism of the silver nanoparticles (AgNPs) on the graphene nanosheets through the dendrimers is shown in Fig. 2. The abbreviation used for graphene modified with a third-generation polyamidoamine dendrimer is G-D, while for graphene-dendrimer-silver nanoparticles it is G-G-Ag.

### Material Characterization

Scanning Electron Microscope, JSM-6610LV, JEOL at 20 kV acceleration voltage equipped with energy-dispersive X-ray spectroscope, Mapping and transmission electron microscope (TEM, FEI Tecnai TF20) were employed to investigate the morphological and microstructural attributes of the synthesized material. The UV-Visible spectra of the graphene and G-D-Ag were recorded on a genesis 10S UV-Vis spectrophotometer (Thermo Scientific), using standard quartz cuvette at room temperature between 250–650 nm. The samples were prepared by dilution the stock solution 4x with distilled water. FT-IR spectra of samples were recorded using a Perkin-Elmer IR spectrophotometer using potassium bromide (KBr) pellets, the pellet was designed by blending the sample and KBr with a ratio of 1:100. The FT-IR measurement was scanned at a range from 400 to 4000 cm^−1^. The He-Ne laser source operating at 0.5 W was utilized for sample excitation.

### Surface-Enhanced Raman Scattering (SERS) spectroscopy

The SERS spectra of samples were obtained by using a Raman spectroscopy system- a Lab Ram HR Evolution Raman spectrometer- equipped with an internal He-Ne 17 mW laser at a 633 nm excitation wavelength. SERS samples were prepared in a small cuvette by using a 4:1 volume ratio of aqueous MTZ solution to G-D-Ag. A 50x objective was used for focusing the laser beam to the solution. The data acquisition time was 20 sec with one accumulation for collection with each SERS spectra. A cuvette with dimensions of 1 cm radius and 2 cm height was used as a sample cell for the Raman spectra. The SERS spectra were obtained in the range from 400–2000 cm^−1^.

### Theoretical Calculations

**Figure 3:**
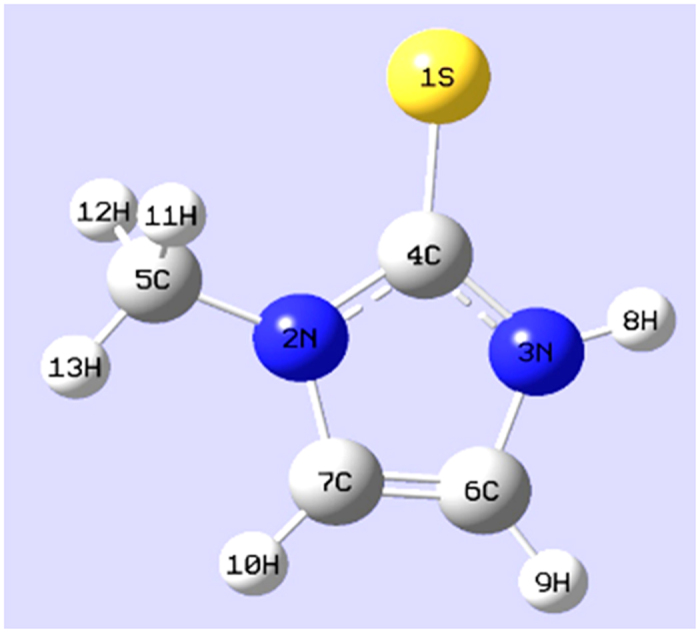
The optimized structure of MTZ.

Density functional theory (DFT) calculations were employed to optimize the structure of MTZ and calculate its vibrational frequencies at the ground level. The Gaussian 09 program was used to carry out the DFT-B3LYP/6-311 ++ G(d,p) level of calculation^22^. Atomic displacements associated with each vibrational mode were carefully inspected using Gauss–View software^23^ and corresponding potential energy distributions (PEDs) were computed with Vida software^24^ in order to provide reliable assignments of the normal Raman, as well as SERS spectra, of MTZ. The minimum-energy structure of MTZ with atom numbering adopted is shown in Fig. 3. The vibrational frequencies were compared to the solid state Raman spectra (Table 1).

## Results and Discussion

### Structural analysis of G-D and G-D-Ag

**Figure 4:**
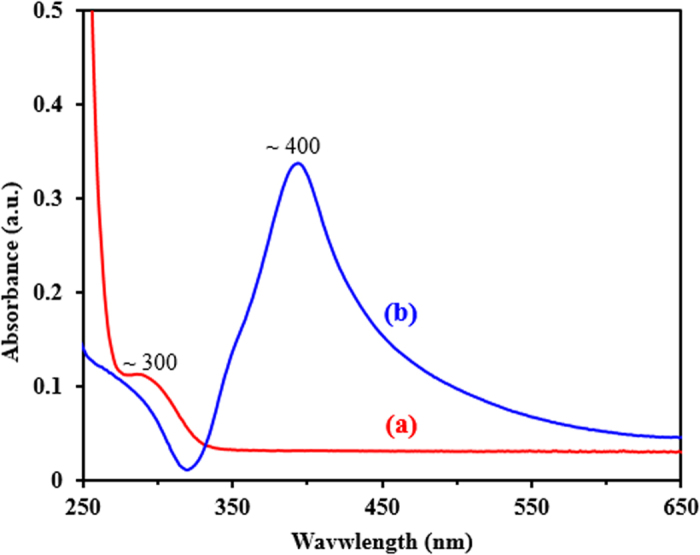
UV-Vis absorption spectra of (**a**) the G-D and (**b**) the G-D-Ag.

The ultraviolet-visible spectra of G-D and G-D-Ag are shown in Fig. 4. The maximum absorption band at 300 nm is attributed to the n-π* electronic transitions of the dendrimer. Moreover, the maximum absorption peak of G-D-Ag is at 400 nm, due to the plasmon resonance of G-D-Ag, indicating the formation AgNPs on the surface of the dendrimer.

**Figure 5:**
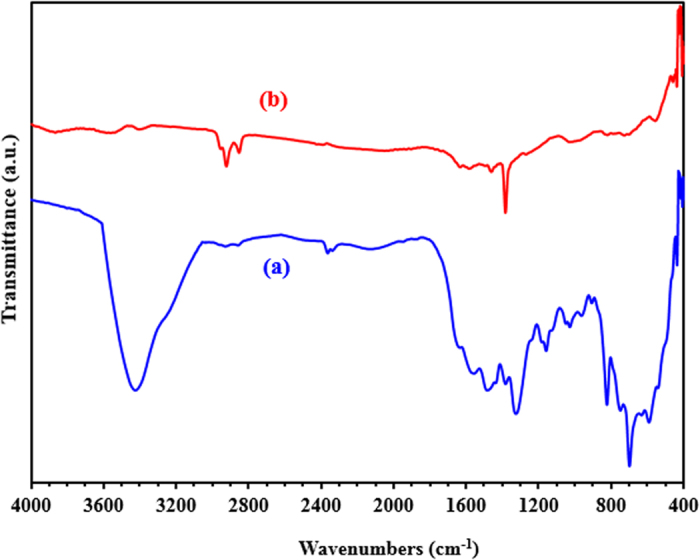
FT-IR spectra of (**a**) G-D and (**b**) G-D-Ag.

FT-IR was employed to confirm the chemical structure of G-D and G-D-Ag. Figure 5 shows the FT-IR spectra of G-D and G-D-Ag. The FT-IR spectrum of G-D shows a weak broadband at ~3418 cm^−1^, corresponding to the vibration of NH_2._ The very low-intensity peaks at 2923 cm^−1^ and at 2854 cm^−1^ are assigned to the symmetric and antisymmetric stretching vibrations of CH_2_, respectively. The bands at 1654 and 1324 cm^−1^ are assigned to C=C and C=O, respectively. The FT-IR spectrum of G-D-Ag differs from that of G-D, as evidenced by the weakening of the NH_2_ band in the range 3350 to 3450 cm^−1^. It suggests that the AgNPs are stabilized in the G-D network through this functional group^25^. The disappearance of the peak, attributed to C-O at 1324 cm^−1^ in the G-D-Ag spectrum, is probably due to the reduction of the oxygenated functional groups through the heat treatment process^15^.

**Figure 6:**
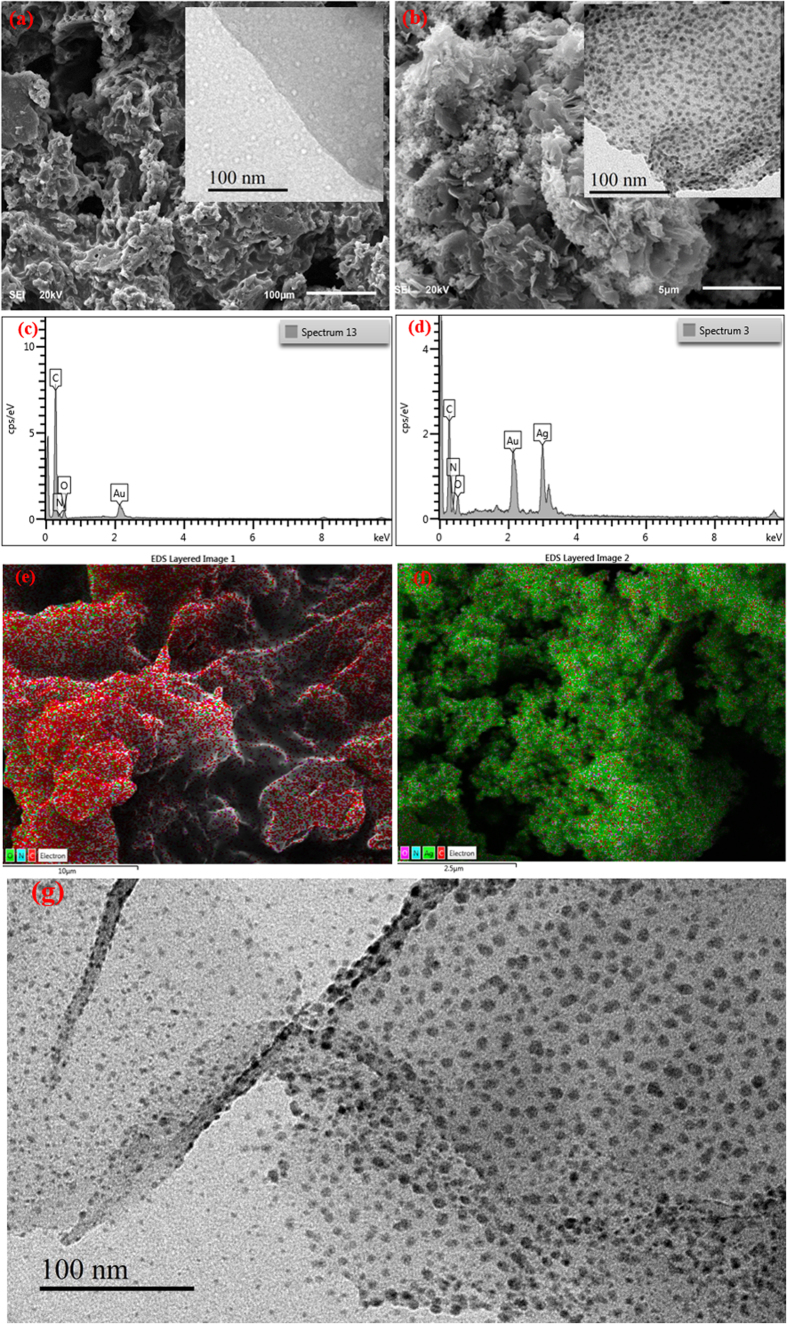
(**a**) Typical SEM image (inset: TEM image) of G-D; (**b**) SEM image (inset: TEM image) of G-D-Ag; (**c**) EDX spectra of G-D; (**d**) EDX spectra of G-D-Ag; (**e**) Mapping image of G-D; (**f**) Mapping image of G-D-Ag; (**g**) TEM image of G-D-Ag.

SEM, EDX and mapping imagings were used as techniques complementary to TEM to investigate the appearance of the synthesized materials, as seen in Fig. 6. The SEM images (Fig. 6a), shows the morphology of the prepared G-D, and the inset TEM image illustrates the formation of multi- dots of dendrimers on the graphene nanosheets. These dots are used as bases, or cores, for attracting and catching the silver ions. The presence of reactive amine groups on the surface of dendrimer-modified graphene was profited to allow the multipoint attachment of the AgNPs through the formation of linkages, (as shown in the mechanism-Fig. 2) which were further transformed to stable secondary amino linkages by reductive treatment with NaBH_4_. This allows for the controlled growth of AgNPs, as shown in the TEM image (Fig. 6g) and the SEM image, with TEM inset (Fig. 6b), which provide evidence that the Ag nanoparticles are well dispersed as a consequence of the stabilization of the growing silver by the different amide groups of the dendrimer. The nanoparticles could be stabilized by interaction with the primary amino groups remaining at the outer surface of the dendrimer. The mapping images, Fig. 6e,f, indicate that the stabilized AgNPs were mostly uniform dispersed. Further characterization was confirmed by EDX spectra (Fig. 6c,d), which confirms the presence of the silver, with strong interaction with the dendrimer, even after washing the sample several times, followed by drying. Therefore, the graphene was successfully used as an indirect support for the silver nanoparticles. The silver nanoparticles were decorated on the dendrimer branches rather than being directly attached to the graphene. This material provides the best SERS enhancement for MTZ compared with the AgNPs loaded graphene, because the dendrimer allows better distribution of AgNPs on the nanosheets, as shown in the TEM image. Therefore, the role of the graphene was as a support; however, the silver nanoparticles were located on the dendrimer branches (linkers) rather directly attached on the graphene. This way the silver nanoparticles were better distributed and decorated on the graphene sheets surface as shown in the TEM image.

### Raman Analysis of G-D and G-D-Ag

**Figure 7:**
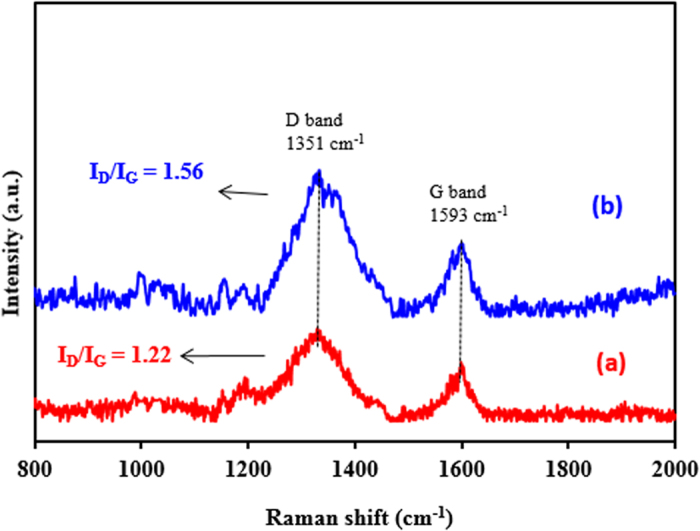
Raman spectra of (**a**) G-D and (**b**) G-D-Ag.

The Raman spectra of the G-D and G-D-Ag are shown in Fig. 7. The Raman spectra of all samples displayed two prominent bands. While the D band around 1350 cm^−1^ is associated with disordered sp^3^ carbon atoms, the G band around 1590 cm^−1^ corresponds to ordered sp^2^–hybridized carbon atoms^26^. Further, the intensity ratio of D and G bands (I_D_/I_G_) increases. The I_D_/I_G_ is used to assess the sp^2^/sp^3^ carbon ratio, which represents the degree of disorder and the average size of the sp^2^ carbon atoms domains. The ratio for G-D-Ag, 1.56, was larger than that for G-D, 1.22, suggesting that more graphitic domains are formed and the sp2 cluster number is increased after introducing the silver via the reduction process. This reflects the functionalization of the AgNPs on the dendrimer-modified graphene^27^. This can be explained by the removal of some oxygen-containing functional groups during the reduction process, leading to the formation of high-level fragmentation along the reactive sites of graphene dendrimer^28^.

### Surface-Enhanced Raman Scattering (SERS) spectra of MTZ with G-D-Ag

**Figure 8:**
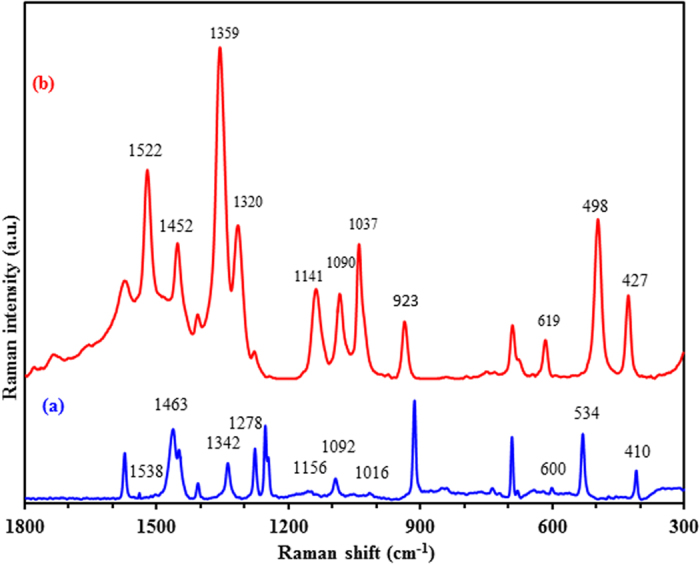
Raman spectrum of (**a**) pure solid MTZ and (**b**) SERS spectrum of 1 × 10^−5^ M MTZ with G-D-Ag as a substrate, Laser ʎ = 633 nm, acquisition time; 20 sec, and objective; 50x.; with the assignments of Raman bands.

The collected Raman spectrum for solid MTZ, compared with a 1 × 10^−5^ M concentration MTZ-(G-D-Ag) SERS spectrum, is depicted in Fig. 8. In order to understand the nature of the interaction between the bounding of the MTZ molecules and the surface of the AgNPs, it is useful to propose proper band assignments for the normal Raman and SERS spectra. For reliable assignments, we conducted DFT assessments of the vibrational frequencies of the single MTZ molecule and compared them with the corresponding ones resulting from the interaction between the silver and MTZ. All these data are listed in Table 1. The DFT method based on the hybrid B3LYP functional and split-valence 6–311 ++ G(d,p) basis set showed good agreement with the experimental results. The band observed at 1342 cm^−1^ and at 1345 cm^−1^ in the solid and solution Raman spectra, respectively, shifted to 1359 cm^−1^ in the SERS spectrum. This band shows the highest enhancement factor. The DFT calculation attributes this band mostly to the N2-C4 stretching (with some contribution from the ring and C6-N3-H bending) and successfully predicts its slight shift to the lower frequency side. Moreover, the modes observed at 1538 and 1463 cm^−1^ have shifted to 1522 and 1452 cm^−1^, respectively, in the SERS spectrum with significant enhancement. PED analysis shows that these bands are associated with S-C and C-N stretching modes (Table 1). The bands at 1278, 1156, 1092, 1016, and 600 cm^−1^ in the normal Raman spectrum are shifted to 1320, 1141, 1090, 1037, and 619 cm^−1^, respectively in the SERS spectrum. These bands show higher intensities in the SERS spectrum.

The Raman bands recorded at 525 and 410 cm^−1^ are attributed to SCN bending and were observed at 498 and 427 cm^−1^, respectively, in the SERS spectrum. The red shift of the former, and the blue shift of the latter SCN bending modes have been reproduced successfully by the DFT approach, which makes it convenient to assign them accordingly. This suggests that in the SERS experiments silver particles interact with the MTZ molecules through both sulfur and nitrogen positions at a comparable level.

### SERS Enhancement Factors of MTZ

The SERS enhancement factors (EFs) for the vibrations of MTZ (1 × 10^−3^ M) on G-D-Ag to the corresponding band obtained from 1.0 M saturated solution were calculated using the following equation.


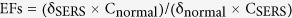


where δ and C are the Raman mode intensity and sample concentrations, respectively. The EFs for the SERS peaks of MTZ on G-D-Ag are given in Table 2. The EFs are not the same for the different MTZ modes; the maximum enhancement was observed at 1342 cm^−1^.

### SERS Spectra of MTZ at Different Concentrations

**Figure 9:**
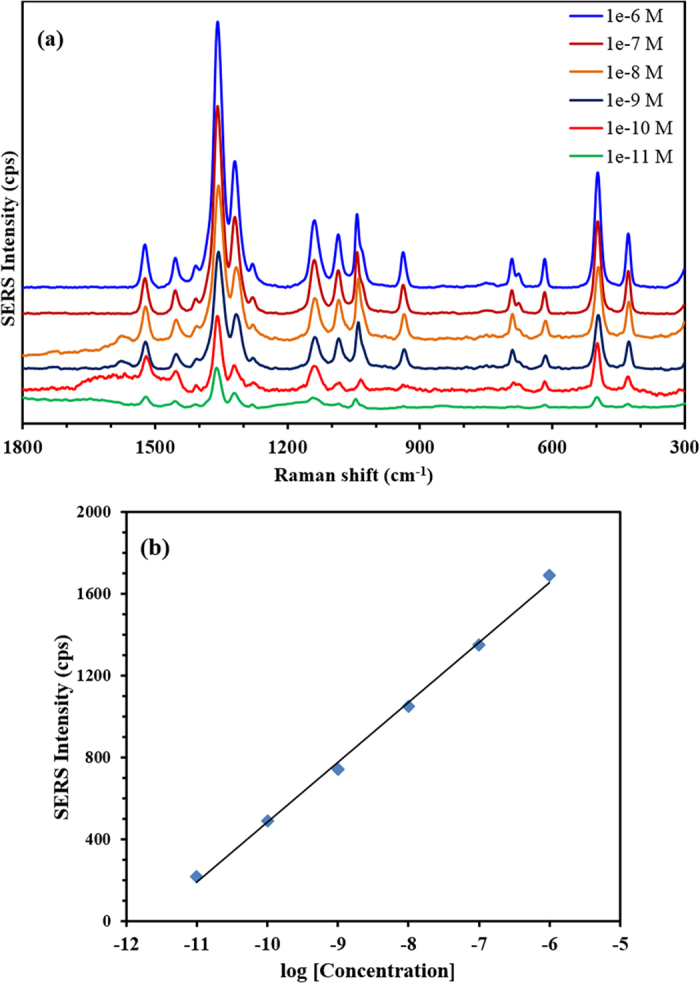
(**a**) SERS spectra of MTZ with different concentration using G-D-Ag, (**b**) calibration curve of the band at 1359 cm^−1^. Laser ʎ = 633 nm, acquisition time; 20 sec, and objective; 50x.

The SERS spectra of MTZ aqueous solution with G-D-Ag as a substrate at different concentrations are given in Fig. 9a. The intensities of the SERS spectra increase with an increase in the concentration of MTZ. This suggests that the SERS intensities are proportional to the molecular quantity of MTZ. The highest enhanced band, at 1359 cm^−1^ in the SERS spectra, was selected for creating a qualitative analysis of MTZ. A plot of the SERS response versus the logarithmical scale of 10^−6^ M to 10^−11^ M of MTZ at 1359 cm^−1^ was obtained, (Fig. 9b), showing a good coefficient of determination (R^2^) of 0.9976. Within the dynamic range, the lowest concentration measured in the SERS analysis of the MTZ solution was 10^−11^ M. To evaluate the analytical performance of the proposed method, parameters such as linearity, repeatability, limits of detection and dynamic range were investigated under optimum experimental conditions. The results of the linear equations, dynamic range, and R^2^ for the obtained calibration curves of MTZ with G-D-Ag substrate are summarized in Table 3.

Good linear relations between the enhanced SERS bands’ intensities in counts per second (cps) and the logarithmical scale of MTZ concentrations were noted with a wide dynamic linear range or linear working range (LWR) for MTZ with the substrate. The precision of the proposed method was checked by replicate analysis of the working standard of MTZ drug at six concentration levels. The relative standard deviation (RSD) for all concentration levels was <2.2%, which indicates both the precision and repeatability of the proposed method. The reproducibility of the method using the same batch of the prepared material was obtained in five days, with a corresponding relative average standard deviation of less than 4%.

The results obtained by the reported method in this study were compared with some methods reported in the literature in terms of calibration range, detection limits, and determination coefficients (R^2^). The comparison with other methods for the determination of MTZ is summarized in Table 4. In comparison to other methods for determination of the MTZ, the proposed method has attracted more interest due to its sensitivity, good dynamic range, and simplicity

### Application of the proposed method for the determination of MTZ in real samples

Determination of MTZ in tablet samples was examined to demonstrate the ability of the SERS method for the determination of MTZ in real samples. The proposed method was applied for the determination of MTZ in the commercial pharmaceutical dosage forms, tablet samples. In order to access the matrix effect, the relative recoveries of the method were calculated. The obtained results, shown in Table 5, indicate the accuracy of the method, as well as the low interference limits caused by the frequently encountered excipients and the degradation products. Thus, the SERS method retained its efficiency for the determination of MTZ in real samples.

## Conclusion

We have reported the synthesis of graphene functionalized with polyamidoamine dendrimer decorated with silver nanoparticles (G-D-Ag), characterized by using various techniques including SEM, TEM, FTIR and UV. The SERS method was exploited to record the vibrational frequencies of MTZ adsorbed on G-D-Ag. The optimized conformation and vibrational assignments of MTZ were carried out using a DFT calculation with a B3LYP/6-311 ++ G (d, p) basis set. The vibration assignments and the wavenumber of vibration frequency bands in the theoretical spectra were in agreement with those of the experimental spectra. Most of the bands related to N and S atom were apparently enhanced and slightly shifted. These results confirm that MTZ molecules were adsorbed on the G-D-Ag, probably through the lone pair on the N and S atoms. The correlation between the logarithmical scale of MTZ concentration and the SERS signal was linear within a dynamic range of 10^−6^–10^−11^ and R^2^ of 0.9976, and with good detection limits down to 1.43 × 10^−12^.

## Additional Information

**How to cite this article**: Saleh, T. A. *et al*. Graphene Dendrimer-stabilized silver nanoparticles for detection of methimazole using Surface-enhanced Raman scattering with computational assignment. *Sci. Rep*. **6**, 32185; doi: 10.1038/srep32185 (2016).

## Figures and Tables

**Table 1:** Infrared, Raman, SERS and calculated DFT vibrational frequencies (cm^−1^) of MTZ.

Observed				Calculated		Assignments with Corresponding potential energy distributions (PEDs) (%)
IR	Raman (Solid)	Raman (Solution)	SERS	MTZ	MTZ-Ag	
3159 w	3161 w	3166 m		3162	3166	97% ν (C7-H)
3104 w	3105 w	3106 vw		3142	3147	98% ν (C6-H)
3012 w				3022	3021	96% ν (C5-H11)
				2999	2995	100% ν (C5-H12)
2949 vw	2950 m	2960 m	2945 m	2936	2932	96% ν (C5-H13)
1578 vs	1579 s	1580 m	1567 w	1588	1581	63% ν (C6 = C7), 10% δ (N3-H) bend
	1538 vw		1522 vs	1509	1496	24% ν (N2-C4), 15% ν (C-C), 38% δ (H11-C-H12) bend
				1473	1467	23% ν (S-C4), 14% ν (C4-N) bend, 10% δ (N3-H) bend,
	1479 vw	1480 vs		1466	1457	72% δ CH_Me_ scissoring
1462 s	1463 vs	1460 vw	1452 s	1459	1452	23% ν (S-C4), 14% ν (N3-C4), 12% δ (C-H) bend,
1403 m	1410 m	1410 vw	1408 w	1415	1411	14% ν (N2-C4), 14% ν (N3-C6), 13% ν (S-C4), 30%δ (C -H) bend
1339 vs	1342 s	1345 s	1359 vs	1315	1328	32% ν (N2-C4), 11% δ ring bend, 19% δ C6-N3-H bend
1274 s	1278 m	1281 m	1320 s	1285	1309	15% ν (N2-C5), 19% δ N3-H (C6-H) bend, 14% δ ring breathing
1248 m	1252 vs	1255 vw	1277 vw	1212	1237	51% ν (N3-C4), 18% δ N3-H (C6-H) bend, 13% δ (C7-H) bend
1152 vs	1156 vw	1153 m	1141 m	1159	1150	16% ν (N3-C6), 16% ν (S-C4), 15% δ (H11-C-H12) rock,
1086 vw	1092 m	1088 vw	1090 m	1089	1091	46% ν (N3-C6), 14%δ (N3-H) bend, 21% δ (C7-H) bend
1014 s	1016 m	1017 vw	1037 m	1013	1022	15% ring CH bend, 13% δ CH_Me_ bend, 41% δ ring bend
913 m	915 vs	916 s	937 w	913	923	12% ν (N2-C4), 12% δ N3-H (C6-H) bend, 62% δ ring bend
818 w	810 vw		830 vw	806	818	89% γ (H-C6-C7-H) twist
673 vs	679 vw	684 vs	687 w	685	699	25% δ (C7-N2-C5) bend, 15% δ (C4-N2-C5) bend
	643 vw		670 vw	650	667	47% ring CH bend, 39% γ (N3-C4-N2)
599 vw	600 vw	602 vw	619 m	603	623	78% γ CN ring bend.
527 vs	525 m	522 w	498 s	534	520	53% δ (S-C4-N3) bend, 25% δ (S-C4-N2),
	493 vw			503	569	84% γ (N3- C6-C7)
411 s	410 s	410 m	427 m	411	421	71% δ (S-C4-N2)
	264 m	260 m	279 w	238	251	85% γ (C4-S) wag
	208 vw	209 vw		207	220	76% γ ring

Values are in cm^−1^; *ν*, stretch; γ, bend; δ, symmetric. vs. very strong; s, strong; m, medium; w, weak; vw, very weak.

**Table 2:** SERS enhancement factor of MTZ on G-D-Ag substarte.

SERS spectra (cm^−1^)	Enhancement Factor (EF)
1522	8.3 × 10^4^
1452	1.1 × 10^4^
1359	1.5 × 10^5^
1320	2.5 × 10^4^
1141	1.0 × 10^4^
1090	2.3 × 10^4^
1037	3.8 × 10^4^
619	1.4 × 10^4^
498	2.0 × 10^4^
427	2.4 × 10^4^

**Table 3:** Regression equation between Raman intensities and concentrations of MTZ and their coefficient of determination (R^2^).

Raman Peaks	Regression Equation	R^2^	Dynamic linear range (M)	LOD*(M)
1359 cm^−1^	y = 292.43x + 3409.8	0.9976	10^−6^–10^−11^	1.43 × 10^−12^
1320 cm^−1^	y = 144.97x + 1651.9	0.9921	10^−6^–10^−11^	2.67 × 10^−12^
498 cm^−1^	y = 124.14x + 1479	0.9744	10^−6^–10^−11^	3.71 × 10^−12^
427 cm^−1^	y = 63.771x + 739.39	0.9651	10^−6^–10^−11^	0.91 × 10^−11^

^*^LOD: limit of detection.

**Table 4:** Comparison of dynamic linear range, detection limits between and coefficient of determination (R^2^) this method and other methods for the determination of MTZ.

Method	Dynamic linear range (M)	Limit of detection (M)	R^2^	Ref.
SERS	10^−6^–10^−11^	See Table 3	See Table 3	Present work
SERS	5.0 × 10^−8^–5.5 × 10^−7^	7.4 × 10^−5^	0.998	29
SERS	1.8 × 10^−9^–1.3 × 10^−6^	8.8 × 10^−10^	0.9992	30
Flow-Injection	1.75 × 10^−5^–8.75 × 10^−4^	8.75 × 10^−6^	0.999	31
Capillary Electrophoresis	1.0 × 10^−7^–2.0 × 10^−4^	5.0 × 10^−8^	0.9995	32
DPV	1.0 × 10^−7^–2.0 × 10^−5^	2.0 × 10^−8^	0.998	33
HPLC	0.2 × 10^−6^–2.0 × 10^−6^	0.18 × 10^−6^	0.9975	34
SWV	6.0 × 10^−6^–240 × 10^−6^	1.98 × 10^−6^	0.9996	35

**Table 5:** Determination of MTZ in pharmaceutical tablet samples (n = 3); Recovered concentrations obtained for MTZ using a SERS method with G-D-Ag and calibration curve at 1359 cm^−1^ (n = 3).

Sample	Expected	Found	Recovery %	Confidence interval	Bias (%)
Tablet 1	5 mg/g	4.93 mg/g	98.6	0.31 × 10^−6^ M	−1.4
Tablet 2	5 mg/g	4.88 mg/g	97.6	0.31 × 10^−6^ M	−2.4
Spiked 1	2.5 × 10^−6^ M	2.61 × 10^−6^ M	104.4	0.48 × 10^−6^ M	+4.4
Spiked 2	5.0 × 10^−6^ M	5.13 × 10^−6^ M	102.6	0.72 × 10^−6^ M	+2.6
